# Load and breath-hold: impact of 7-day creatine monohydrate loading on breath-hold exercise performance — a double-blind randomized controlled trial

**DOI:** 10.1080/15502783.2026.2732743

**Published:** 2026-10-13

**Authors:** Natalija Kurmazovic, Tzortzis Nomikos, Manolis Adamakis, Scott C. Forbes, Giannis Arnaoutis, Brizita Djordjevic, Vanja Todorovic, Periandros Kritsiligkos

**Affiliations:** a Department of Bromatology, Faculty of Pharmacy, University of Belgrade, Belgrade, Serbia; b Department of Nutrition and Dietetics, School of Health Sciences & Education, Harokopio University, Kallithea, Greece; c School of Physical Education and Sport Science, National and Kapodistrian University of Athens, Athens, Greece; d Department of Physical Education Studies, Faculty of Education, Brandon University, Brandon, Manitoba, Canada; e Independent Researcher, Athens, Greece

**Keywords:** Breath-hold, creatine monohydrate, loading, performance, anaerobic endurance

## Abstract

**Background:**

Creatine supplementation enhances anaerobic energy availability and buffering capacity; however, its impact on muscle work under voluntary breath-hold (BH) remains unexplored. BH exercise increases reliance on anaerobic ATP production and creates a metabolic environment in which phosphocreatine-derived ATP may be particularly valuable. The aim of this study was to determine whether creatine monohydrate loading improves BH exercise performance in trained BH divers.

**Methods:**

A double-blind, randomized, placebo-controlled study assessed the effects of a 7-day creatine monohydrate loading protocol (20 g/day) on performance outcomes and fatigue markers during BH exercise performed on a bicycle ergometer in 20 trained BH divers (18 males, 2 females; mean age 41.6 ± 8.6 years). The trial was registered at ClinicalTrials.gov (Identifier: NCT07233707) on 16th November 2025 under the title Load and Hold: Impact of 7-Day Creatine Monohydrate Loading on Breath-Hold Cycling Performance (BREATHE-C). Testing consisted of a brief warm-up followed by a maximal cycling trial performed on a single breath, during which participants aimed to cover the greatest possible distance. Following pre-supplementation testing, participants were randomly assigned to either the creatine monohydrate (CR) or the placebo (PL) group. Pre- and post-supplementation measurements included total work (TW), time to exhaustion (TTE), peripheral blood lactate accumulation (LA), mean partial pressure of carbon dioxide (pCO₂) in exhaled air following BH exercise, and ratings of perceived exertions for BH (RPE-BH) and muscle fatigue (RPE-MF). In addition, participants underwent dual-energy X-ray absorptiometry to assess changes in body composition.

**Results:**

Significant Time × Group interactions were observed for TW (*p* = 0.011), TTE (*p* = 0.028), and fat-free mass (*p* = 0.017), with greater changes observed following creatine supplementation compared to PL. Furthermore, there was a significant Time × Group interaction for LA (*p* = 0.003), with greater changes observed in the PL group compared to CR. No significant between-group differences were observed for pCO₂, RPE-BH, RPE-MF, fat mass, or total body mass.

**Conclusions:**

Creatine enhanced performance, as reflected by increases in TW and TTE, without elevating RPE-BH, although a slight increase in RPE-MF was observed. LA was lower in the CR compared to PL group, despite greater performance improvements. Overall, creatine appears to enhance BH exercise performance in trained BH divers, suggesting a potentially effective ergogenic strategy for BH-related sports.

## 1. Introduction

Creatine, synthesized from arginine, glycine, and methionine and stored predominantly in skeletal muscle, increases intramuscular phosphocreatine availability, thereby enhancing rapid ATP re-synthesis and improving hydrogen ion buffering capacity [1]. Creatine has become one of the most popular and widely studied ergogenic aids [2]. During maximal intensity efforts, the ATP–PCr system is the most prominent energy system used to replenish ATP for approximately the first 5–10 seconds of exercise before the relative contribution shifts toward glycolytic and oxidative energy systems [3]. Therefore, its ergogenic benefits have been shown to enhance performance during short, intense bouts of exercise or activities involving repeated high-intensity efforts, leading to improvements in work capacity, power output, strength, and anaerobic threshold [1,2,4].

Breath-hold diving (BHD) is a non-Olympic sport comprising several disciplines that can be categorized as static, dynamic, and depth. Static disciplines involve holding the breath for as long as possible while remaining stationary, whereas dynamic disciplines involve covering the greatest possible distance underwater, and depth disciplines involve descending to the greatest possible depth on a single breath. These disciplines place high demands on oxygen conservation, as well as tolerance to rising CO₂ and hydrogen ions. Consequently, BH divers train to optimize oxygen utilization and enhance tolerance to metabolic by-products caused by the shift toward anaerobic metabolism, which leads to increased lactate production [5,6]. However, it should be noted that no form of exercise is purely anaerobic or aerobic; all three energy systems, anaerobic alactic, anaerobic lactic, and aerobic, contribute to ATP re-synthesis, with their relative contributions depending on the intensity and duration of the exercise [1]. Despite this shift toward anaerobic metabolism, BHD resembles endurance sports due to its sustained, repetitive muscular effort performed at low to moderate intensity.

The literature on creatine supplementation in endurance exercise is heterogeneous, with studies reporting benefits, no effects, or decrements in performance, likely due to differences in supplementation protocols, exercise models, individual responsiveness, and creatine-induced body mass gains [1,7,8]. Moreover, the ergogenic potential of creatine appears to diminish as exercise duration increases [2]. Although BHD lacks high movement velocities and elevated heart rates, characteristic of sports in which creatine's effects are well established, its relatively short duration and pronounced anaerobic demands may provide a favorable context for creatine supplementation. For example, a 100–300 m dynamic BH dive is typically completed in approximately 1–4 minutes, during which anaerobic alactic energy provision and buffering capacity may influence performance. Additionally, potential weight gain from creatine loading is unlikely to impair performance, as BHD is a non-weight-bearing activity.

Theoretically, creatine monohydrate supplementation could enhance BHD performance by supplying an anaerobic energy source that is independent of oxygen availability and providing an energy substrate that does not generate lactate and hydrogen ions. As such, a BH exercise protocol designed to mimic metabolic demands and intensity of a maximal dynamic BH dive with fins, was used in the present study to investigate the effects of creatine monohydrate loading on BH exercise performance on a bicycle ergometer in trained BH divers. To date, no study has examined creatine monohydrate supplementation ergogenic effects in trained BH divers, so this study represents a novel and physiologically relevant context in which to evaluate creatine's ergogenic potential.

We hypothesized that creatine supplementation will (1) enhance BH exercise performance by increasing total work (TW) and time to exhaustion (TTE), (2) delay metabolic acidosis, as reflected by reduced lactate accumulation (LA), (3) decrease in partial pressure of carbon dioxide (pCO₂) in exhales following BH exercise, and (4) reduce rating of perceived exertion (RPE), as measured by the Borg scale [9,10]. Additionally, we assessed body composition pre- and post-supplementation in order to characterize potential confounding effects associated with creatine loading.

## 2. Materials and methods

### 2.1. Participants

Trained BH divers, defined as individuals with at least one year of BHD training experience, were recruited for this randomized, double-blind, placebo-controlled trial. Eligibility criteria included regular BHD training within the 12 months preceding enrollment and a minimum of one year of competitive experience. Due to the limited population size, a convenience sampling approach was used and no a priori power analysis was performed. Additionally, individuals were excluded if they had used creatine monohydrate within the six weeks prior to the study enrollment [11].

The trial was registered at ClinicalTrials.gov (Identifier: NCT07233707). All procedures were approved by the Institutional Ethics Committee (approval number: 3301/21-07-2025) and performed in accordance with the Declaration of Helsinki. All participants received written and oral information regarding the study procedures and provided written informed consent prior to participation. They were informed that participation was voluntary, uncompensated, and that they could withdraw from the study at any time without consequence.

### 2.2. Study design

The study consisted of one pool session and four laboratory visits. The laboratory sequence included: (1) a graded exercise test (GXT), (2) a familiarization session, (3) a pre-supplementation experimental trial, and (4) a post-supplementation experimental trial. Visits 1 and 2 were used to develop and establish the validity and reliability of the BH exercise protocol and to facilitate participant pairing for randomization. The detailed development, validity, and reliability outcomes are reported in a separate publication; however, a brief overview of the full study design is provided here for context (Figure 1).

**Figure 1.**
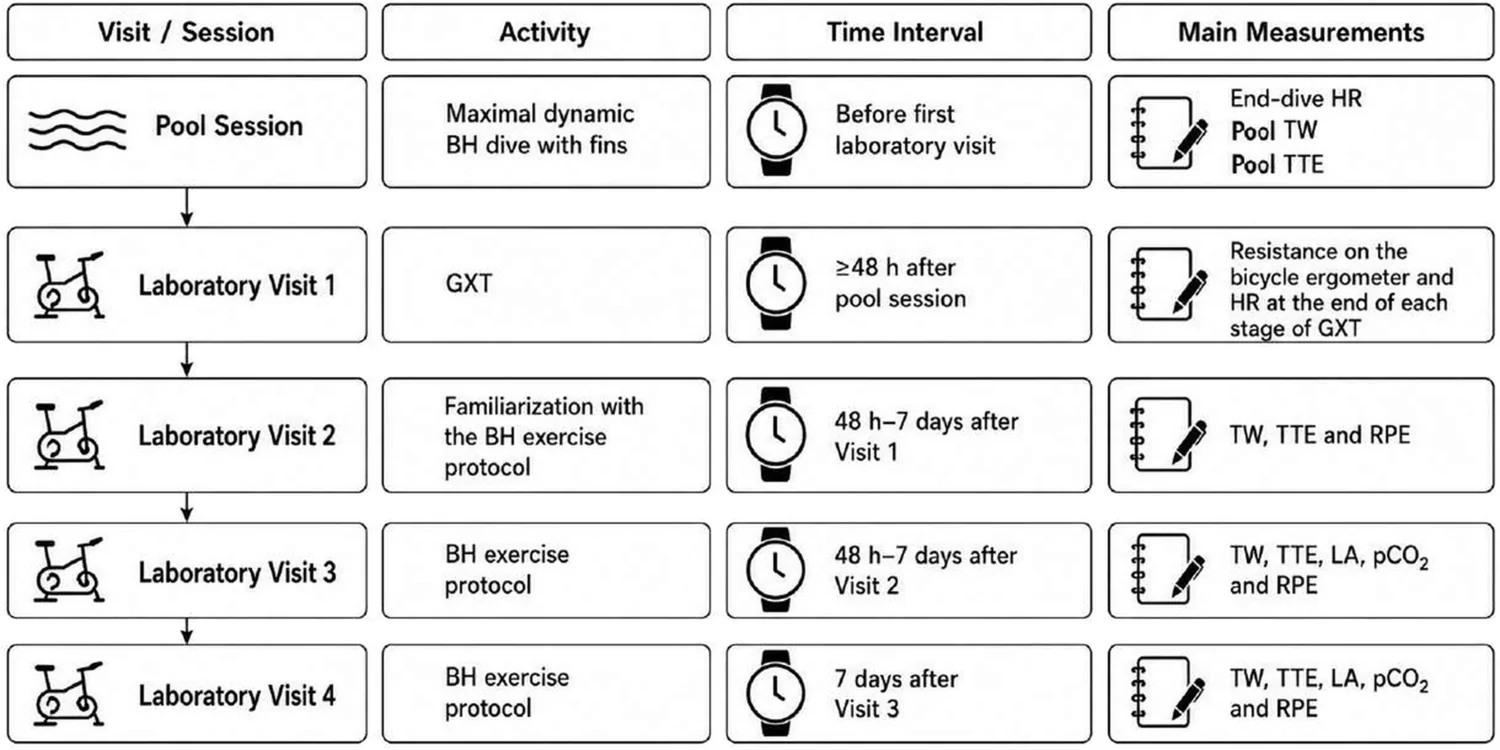
Overview of the study design.

Visits 1–3 were separated by a minimum of 48 hours and a maximum of 7 days, whereas Visits 3 and 4 were separated by the 7-day supplementation period. In addition, visits 2–4 were all scheduled in the morning hours, participants were asked to arrive fasted and abstain from caffeine intake, and visits were scheduled at the same time of day (±1 hour).

Prior to the first laboratory visit, each participant's end-dive HR was measured in a pool immediately following their maximal dynamic BH dive with fins. HR was recorded using an optical HR sensor polar verity sense. The first HR value displayed by the sensor immediately upon surfacing was recorded and later used as the indicator of exercise intensity for the development of the individualized BH exercise protocol. In addition, the distance covered during the dive (pool TW) and total dive duration (pool TTE) were recorded.

#### 2.2.1. Visit 1: graded exercise test

Participants completed a GXT on a Monark Ergomedic 839E bicycle ergometer with the use of MGC Diagnostics Ultima Series™ gas exchange analysis GX system, which were used for all visits. Metabolic measurements obtained using the gas analyzer were configured for breath‑by‑breath analysis of oxygen uptake (VO₂), carbon dioxide production (VCO₂), and respiratory exchange ratio (RER).

Before each session, the gas analyzer was calibrated according to manufacturer protocols. Flow calibration was performed with a 3 L calibration syringe, and gas analyzers for O₂ and CO₂ were calibrated using precision reference gas mixtures (e.g. 5% CO₂ and 12% O₂ in nitrogen). Calibration was performed immediately prior to testing to ensure accuracy.

During the GXT, participants breathed through a facemask connected to the system, and oxygen and carbon dioxide concentrations were recorded continuously. RER was calculated as the ratio of VCO₂ to VO₂ from these breath‑by‑breath data. HR and ergometer resistance were recorded at the end of each stage. Subjects were asked to maintain a cadence of 70–80 rpm throughout the test. Resistance was increased incrementally until participants met at least two of the following criteria: RER ≥ 1.1, RPE ≥ 19, a plateau in VO₂ with an increase in workload, or attainment of age‑predicted maximum heart rate (220 − age). The maximal test was selected because, despite relatively low end-dive HR values, completing all stages to exhaustion provided more data points, enabling a more precise curve and a more accurate estimation of resistance.

Individualized exercise intensity for the BH exercise protocol was determined by plotting HR against resistance on the bicycle ergometer from the GXT and identifying the ergometer resistance corresponding to each participant's recorded end-dive HR. This individualized workload was subsequently used in Visits 2–4.

#### 2.2.2. Visit 2: familiarization session

Participants completed a familiarization trial at their predetermined individualized resistance. The standardized continuous warm-up consisted of: 1 minute of cycling, a 15-second BH while cycling, 1 minute of cycling, a 30-second BH while cycling, and a final 1 minute of cycling. This was followed by a 5-minute rest and a 3-minute countdown, during which participants remained seated in a quiet environment while the time was counted down from 3:00 to 0:00 to prepare them for the maximal BH attempt.

Participants then performed a maximal BH exercise attempt on the bicycle ergometer, instructed to cover the greatest possible distance on a single breath following a maximal inspiration. A mouthpiece connected to a metabolic gas analyzer was used to determine the onset and termination of the BH, and nose clips ensured no air leakage. Any attempt to breathe would require exhalation, which would be detected by the gas analyzer, whereas oral air topping-up would be readily audible and visually apparent. Participants were required to maintain their self-selected pace within  ± 5 RPM of their target on all visits, with a minimum cadence of 50 RPM.

Distance covered during a maximal BH exercise attempt (total work, TW), BH duration (time to exhaustion, TTE), and ratings of perceived exertion for both BH and muscle fatigue (RPE-BH and RPE-MF) were recorded during this visit to pair participants based on performance and create balanced groups.

#### 2.2.3. Visits 3: pre-supplementation experimental trial

On Visit 3, participants completed the same standardized BH exercise protocol as in Visit 2. In addition to TW, TTE, RPE-BH and RPE-MF, earlobe capillary blood lactate concentration was measured. Blood lactate was measured using a portable analyzer Lactate Scout 4, which provides results within approximately 10 seconds. The first lactate measurement (L1) was obtained following a 5-minute seated rest period preceding the 3-minute countdown prior to the maximal attempt, and the second measurement (L2) was taken within 15 seconds after completion of the maximal effort in the same position. Lactate accumulation (LA) was calculated as the difference between L2 and L1.

Before the trial, whole-body composition was assessed in order to characterize potential confounding effects associated with creatine loading. It was assessed using dual-energy X-ray absorptiometry Lunar iDXA. All DXA scans were performed in accordance with the manufacturer's guidelines and were conducted by a trained technician to ensure precision and reliability. Participants were positioned supine during the scan, and measurements were taken under controlled conditions, including consistent temperature and low ambient light, to minimize variability. Quality control procedures were performed prior to each scanning session to verify device calibration and ensure the accuracy of measurements.

#### 2.2.4. Visit 4: post-supplementation experimental trial

Following the supplementation period, participants returned for post-supplementation testing, which mirrored the procedures conducted during Visit 3. Participants completed the standardized BH exercise protocol, with TW, TTE, and RPE recorded. Capillary blood lactate measurements (L1 and L2) were collected at the same time points as pre-supplementation. Body composition was reassessed via DXA.

### 2.3. Supplementation protocol

Participants followed a creatine loading protocol, taking either creatine monohydrate (Creapure® Alzchem Trostberg GmbH, 5 g per dose) or placebo (maltodextrin, 5 g per dose) four times daily (total: 20 g/day) for seven consecutive days. A 7-day loading period (within the typical 5–7 day range) was selected to ensure adequate muscle saturation [11], as muscle biopsies to confirm creatine levels were not performed. Supplements were provided in identical, unlabeled sachets. Participants were instructed to dissolve each dose in water and consume it evenly distributed throughout the day. Compliance was monitored via verbal confirmation during Visit 4.

### 2.4. Pairing, randomization and blinding

After the pre-supplementation trial (Visit 3), participants were randomly assigned to either the creatine (CR) or placebo (PL) group using a matched-pair procedure. Participants were first paired based on (1) sex and (2) individual cycling resistance used during the BH protocol. When more than two participants had the same or similar values for these two criteria, pairing was further refined sequentially according to (3) performance during the pre-supplementation test, (4) age, and (5) body composition. Body composition pairing was initially performed using body mass index (BMI); however, groups were later compared for baseline differences in total body mass (TBM), fat-free mass (FFM), and fat mass (FM) derived from DXA results. Within each matched pair, one participant was allocated to the CR group and the other to the PL group by an investigator not involved in participant recruitment, testing, or data analysis.

The study was conducted in a double-blind manner. Participants, investigators involved in testing, outcome assessment, and data analysis remained blinded to group allocation until completion of data collection and primary analyzes. Creatine and placebo supplements were identical in appearance, taste, and packaging, ensuring maintenance of blinding throughout the study.

### 2.5. Measurements and data collection

Performance outcomes included TW measured in meters and TTE measured in seconds. As an objective fatigue marker, peripheral blood LA (L2-L1) was assessed from the earlobe before (L1) and after (L2) the BH exercise. Partial pressure of CO₂ (pCO₂) was assessed from exhaled air collected following BH exercise. For each participant, all breaths recorded up to the point of peak pCO₂ were included in the analysis. The mean pCO₂ value was calculated by summing the pCO₂ values of these breaths and dividing by the total number of breaths. Subjective fatigue was evaluated using RPE, assessing muscular fatigue (related to metabolic acidosis) and BH discomfort (related to CO₂ accumulation) following task cessation. Body composition was measured using DXA during Visits 3 and 4 to determine any change in TBM, FFM, and FM. In addition, a 24-hour dietary recall and 7-day training data were collected before visits 2–4 to monitor and confirm the stability of dietary intake and training type and volume throughout the study period. Participants self-reported type, duration, and frequency of training sessions, as well as all foods and beverages consumed during the 24 hours prior to laboratory visits, including the amounts consumed. Dietary intake data were subsequently analyzed for total energy intake using Soma Nutri software.

### 2.6. Statistical analysis

Descriptive statistics, including means and standard deviations, were calculated for all participant characteristics and outcome measures. Baseline comparisons between groups were conducted using independent-samples t-tests for age, BMI, TBM, FFM, FM, and resistance on the bicycle ergometer. As baseline resistance values significantly influenced baseline performance, all baseline performance comparisons were subsequently analyzed using ANCOVA with resistance as a covariate.

To evaluate the effects of creatine versus placebo supplementation, two-way repeated-measures ANOVAs were conducted for all supplementation outcomes, as well as for the nutritional and training data, examining the main effects of Time, Group, and the Time × Group interaction. For variables potentially influenced by changes in body composition, repeated-measures ANOVA was additionally performed with body-composition variables that differed significantly between groups, included as covariates. Post hoc analyzes with Bonferroni correction were conducted only for variables that demonstrated a statistically significant Time × Group in order to control for Type I error across multiple comparisons.

All statistical analyzes were performed using SPSS version 29.0 for Windows (IBM SPSS Corp., Armonk, NY, USA), with significance set at *p* < 0.05. Effect sizes were reported where appropriate to quantify the magnitude of observed effects. Cohen d and partial η^2^ were estimated as a measure of effect size for t-tests and F-Tests, respectively. Cohen's d value of 0.20 was associated with a small effect, 0.50 with a medium effect, and 0.80 or greater with a large effect [12]. Moreover, a partial η^2^ value between 0.01 and 0.06 was associated with a small effect, between 0.06 and 0.14 with a medium effect, and 0.14 or greater with a large effect [13].

An intention-to-treat (ITT) analysis was performed including all randomized participants, irrespective of protocol adherence or subsequent exclusion. ITT results were compared with those of the per-protocol analysis to assess the robustness of the findings.

## 3. Results

### 3.1. Participant characteristics

A total of 20 participants completed all phases of the study. Table 1 summarizes participants' characteristics and pool session measurements for the total sample and by group.

**Table 1.** Participants' characteristics and pool session measurements.

Variable	Creatine	Placebo	Total sample
Sex (M/F)	9/1	9/1	18/2
Age (years)	41.7 ± 8.3	41.6 ± 9.4	41.6 ± 8.6
BHD training experience (years)	4.4 ± 2.7	4.5 ± 3.6	4.45 ± 3.2
Body mass index (kg/m^2^)	26.6 ± 2.1	25.7 ± 3.3	26.1 ± 2.8
Pool TW (m)	118.9 ± 44.8	112.8 ± 25.1	115.9 ± 36.5
Pool TTE (s)	115.0 ± 43.7	108.1 ± 30.3	111.6 ± 37.8
End-dive HR (bpm)	90.4 ± 13.1	88.9 ± 12.1	89.7 ± 12.6

Initial baseline analyzes included comparisons of BMI and age between groups, both of which showed no significant differences at baseline (BMI: t(18) = 0.73, *p* = 0.47, Cohen's d = 0.33; age: t(18) = 0.57, *p* = 0.58, Cohen's d = 0.25).

### 3.2. Supplementation side effects and compliance

The study was completed with no dropouts. No adverse effects were reported in the CR group, and one participant in the PL group noted mild bloating that did not affect participation. Creatine stores and supplementation compliance were not directly measured; however, participants confirmed full compliance with the instructed dosing schedule and returned all empty supplement sachets at the end of the intervention.

One participant from the CR group was excluded from the final analysis for non-compliance with two prespecified criteria: (1) failure to achieve the target cadence established during visit 2 within ±5 RPM while remaining above 50 RPM; and (2) failure to maintain training regimen during the supplementation period. These deviations likely contributed to reduced TW, TTE, and LA during post-supplementation testing, potentially attenuating between-group differences in TW and TTE, exaggerating the difference in LA, and potentially affecting body composition outcomes. The final sample comprised 19 participants (17 males and 2 females). Figure 2 shows a flow diagram of the progress through the phases of the trial for the two groups.

**Figure 2.**
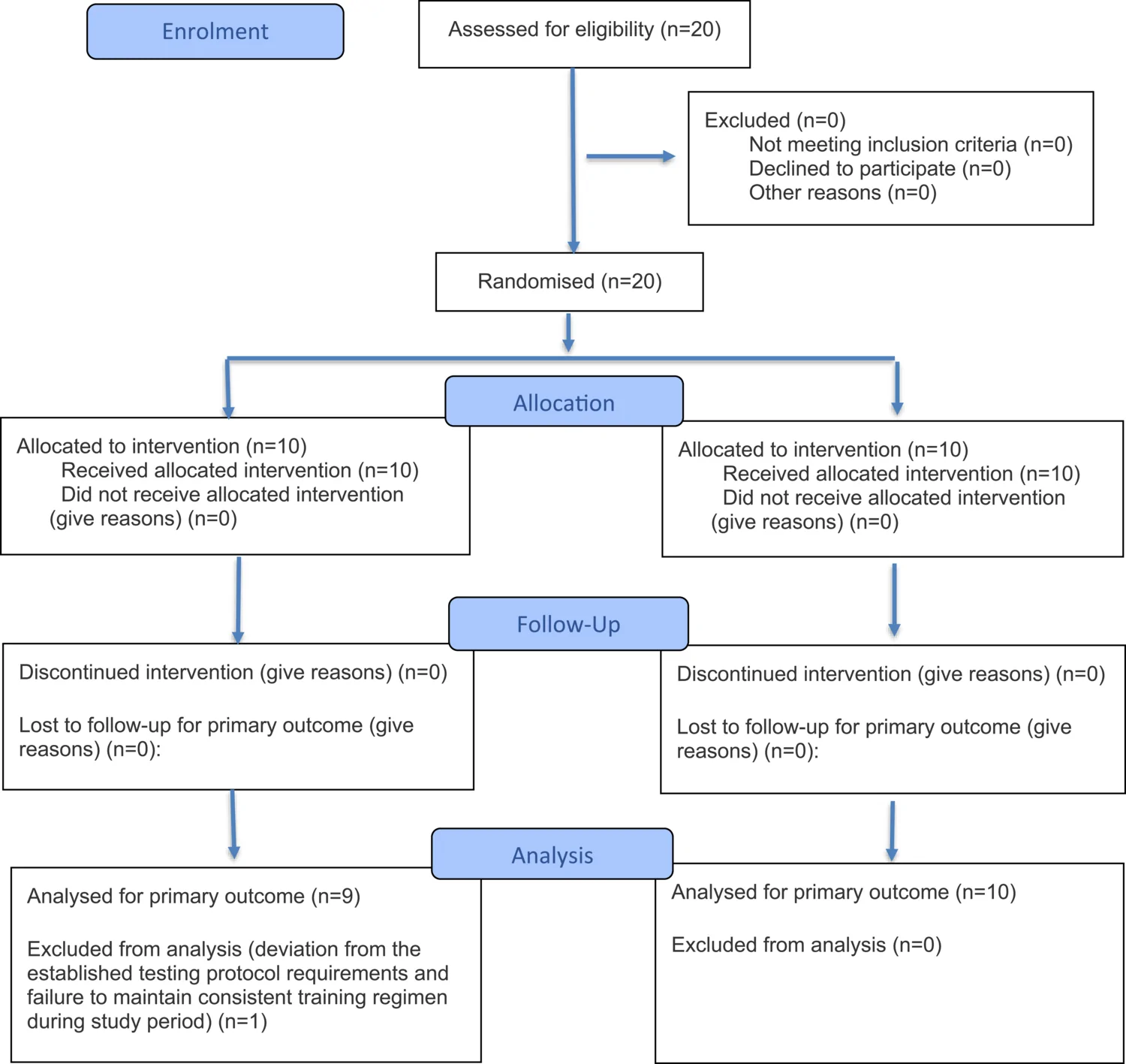
CONSORT flow diagram. Flow diagram of the progress through the phases of a randomized trial of two groups (that is, enrollment, intervention allocation, follow-up, and data analysis). Citation: Hopewell S, Chan AW, Collins GS, Hróbjartsson A, Moher D, Schulz KF, et al. CONSORT 2025 Statement: updated guideline for reporting randomized trials. BMJ. 2025;388:e081123. https://dx.doi.org/10.1136/bmj-2024-081123. © 2025 Hopewell et al. This is an Open Access article distributed under the terms of the Creative Commons Attribution License (https://creativecommons.org/licenses/by/4.0/), which permits unrestricted use, distribution, and reproduction in any medium, provided the original work is properly cited.

### 3.3. Baseline group comparisons

Baseline group comparisons of ergometer resistance, performance, age, and body composition (TBM, FFM, and FM) were conducted again after exclusion of the two previously mentioned participants.

CR group had a mean resistance of 38.33 ± 39.89 Watts, while PL group showed a mean resistance of 31.67 ± 32.77 Watts. No statistical significant difference was observed between groups (t(16) = 0.39, *p* = 0.704, Cohen's d = 0.18).

Ergometer resistance (included as a covariate) significantly influenced baseline performance (F(4,12) = 4.88, *p* = 0.014, η^2^ = 0.619). There was no overall difference in baseline performance between groups, F(4,12) = 1.80, *p* = 0.193, η^2^ = 0.375.

While TW, RPE-BH, and RPE-MF did not differ significantly between groups at baseline, with group effects of F(1,15) = 0.98, *p* = 0.338, η^2^ = 0.061; F(1,15) = 1.26, *p* = 0.280, η^2^ = 0.078; and F(1,15) = 0.18, *p* = 0.674, η^2^ = 0.012, respectively, TTE differed significantly between groups, F(1,15) = 5.45, *p* = 0.034, η^2^ = 0.266, even after adjusting for resistance. Resistance was a significant predictor for TTE, F(1,15) = 6.78, *p* = 0.020, η^2^ = 0.311, and TW, F(1,15) = 19.33, *p* < 0.001, η^2^ = 0.563, but not for RPE-BH, F(1,15) = 4.48, *p* = 0.051, η^2^ = 0.230, or RPE-MF, F(1,15) = 3.39, *p* = 0.085, η^2^ = 0.184.

Groups did not differ significantly in age at baseline (t(16) = 0.77, *p* = 0.227, Cohen's d = 0.36). No significant differences between groups have been observed at baseline for TBM (t(9.66) = 0.99, *p* = 0.346, Cohen's d = 0.47), FFM (t(16) = 1.07, *p* = 0.154, Cohen's d = 0.51), or FM (t(11.43) = 0.47, *p* = 0.649, Cohen's d = 0.22), indicating that the groups were comparable in these variables at baseline measurement.

### 3.4. Supplementation outcomes

The supplementation outcomes are presented in Table 2. Significant Time × Group interactions were observed for TW, TTE, and LA (Table 2). No significant effects were observed for either mean pCO₂ or RPE-BH, including the effects of Time, Group, or the Time × Group interaction (*p* > 0.05). For RPE-MF, the effect of Group and Time × Group interaction were not significant (*p* > 0.05), however a significant main effect of Time was observed (*p* = 0.033) (Table 2).

**Table 2.** Pre- and post-supplementation outcomes by group.

Variable	Group	Pre (Mean ± SD)	Post (Mean ± SD)	*p* value (Time × Group)	η^2^ (Time × Group)
TW (m)	CR	724.00 ± 246.37	964.56 ± 357.15	0.011	0.322
	PL	745.30 ± 283.83	846.80 ± 262.14		
TTE (s)	CR	118.78 ± 43.78	149.22 ± 52.66	0.028	0.253
	PL	89.50 ± 32.97	101.50 ± 37.13		
LA (mmol/L)	CR	1.17 ± .50	1.22 ± .76	0.003	0.423
	PL	1.23 ± 1.03	2.13 ± 1.10		
Mean pCO₂ (mmHg)	CR	29.27 ± 4.46	30.65 ± 4.83	0.196	0.096
	PL	27.50 ± 5.71	27.62 ± 5.84		
RPE-BH	CR	16.00 ± 2.78	16.11 ± 3.44	0.785	0.005
	PL	16.10 ± 2.03	16.10 ± 2.13		
RPE-MF	CR	13.89 ± 2.47	14.33 ± 2.87	0.443	0.035
	PL	13.10 ± 2.56	14.00 ± 2.98		
TBM (kg)	CR	88.96 ± 5.24	89.20 ± 5.12	0.398	0.042
	PL	84.13 ± 15.46	83.97 ± 14.92		
FFM (kg)	CR	61.83 ± 4.01	62.53 ± 4.04	0.017	0.290
	PL	58.53 ± 9.21	58.24 ± 8.87		
FM (kg)	CR	23.73 ± 4.21	23.30 ± 4.12	0.098	0.153
	PL	22.54 ± 8.43	22.58 ± 8.30		

No significant effects were observed for either TBM or FM, including the effects of Time, Group, or the Time × Group interaction (*p* > 0.05). FFM showed no significant main effects of Group or Time (*p* > 0.05), but a significant Time × Group interaction was observed (Table 2).

Post hoc analysis showed a significant increase in TW and TTE for both groups, while LA increased significantly only in the PL group. Additionally, a significant change was observed for FFM in the CR group (Table 3).

**Table 3.** Bonferroni-adjusted post Hoc comparisons of pre- and post-supplementation changes in outcomes across groups.

Variable	Group	Wilks' Λ	F(1,17)	*p*	η^2^	Mean difference (standard error)	95% CI
TW	CR	0.271	45.823	<0.001	0.729	240.556 (35.536)	165.580–315.531
	PL	0.652	9.064	0.008	0.348	101.500 (33.713)	30.372–172.628
TTE	CR	0.363	29.803	<0.001	0.637	30.444 (5.577)	18.679–42.210
	PL	0.768	5.145	0.037	0.232	12.000 (5.291)	0.838–23.162
LA	CR	0.994	0.103	0.753	0.006	0.056 (0.173)	−0.310–0.421
	PL	0.362	29.920	<0.001	0.638	0.900 (0.165)	0.553–1.247
FFM	CR	0.721	6.590	0.020	0.279	0.696 (0.271)	0.124–1.267
	PL	0.931	1.264	0.277	0.069	−0.289 (0.257)	−0.831–0.253

When change in TW was adjusted for changes in FFM, a significant main effect of Time remained significant, F(1, 16) = 41.875, *p* < 0.001, partial η^2^ = 0.724. Nevertheless, neither the Time × FFM difference interaction, F(1, 16) = 0.439, *p* = 0.517, partial η^2^ = 0.027, nor the adjusted Time × Group interaction, F(1, 16) = 3.986, *p* = 0.063, partial η^2^ = 0.199, were significant.

### 3.5. Training and nutritional data across visits 3–4

Training and nutritional data for the 7 days preceding the pre- and post-supplementation testing trials are presented in Table 4. No statistically significant main effects of Group or Time were observed for BHD, swimming, or resistance training sessions (*p* > 0.05). For energy intake, the main effect of Group was not statistically significant, whereas a significant main effect of Time was observed, F(1,17) = 11.095, *p* = 0.004, η^2^ = 0.395. The Time × Group interaction effects for all variables are presented in Table 4.

**Table 4.** Training and nutritional data during the 7 days preceding pre- and post-supplementation testing trials.

Variable	Pre CR group	Pre PL group	Post CR group	Post PL group	Time × Group interaction
BHD training sessions (h/week)	2.00 ± 1.20	1.70 ± 0.95	1.87 ± 1.13	1.70 ± 0.95	F(1,17) = 0.162, *p* = 0.693, η^2^ = 0.010
Resistance training sessions (h/week)	0.67 ± 0.71	0.60 ± 1.08	0.90 ± 0.60	0.30 ± 0.68	F(1,17) = 6.008, *p* = 0.025, η^2^ = 0.261
Swimming training sessions (h/week)	1.22 ± 1.79	0.70 ± 1.06	0.60 ± 1.67	1.10 ± 1.52	F(1,17) = 7.389, *p* = 0.015, η^2^ = 0.303
Rest days (*n*)	3.44 ± 1.74	4.00 ± 1.41	3.90 ± 1.62	3.90 ± 1.52	F(1,17) = 1.819, *p* = 0.195, η^2^ = 0.097
Energy intake (kcal/day)	1928.67 ± 345.50	2222.60 ± 755.09	1937.90 ± 365.42	2079.80 ± 777.55	F(1,17) = 14.370, *p* = 0.001, η^2^ = 0.458

Post-hoc analysis showed that, for resistance training, the CR group had a mean difference of 0.222 (SE = 0.157; 95% CI [−0.104, 0.548]; *p* = 0.169), whereas the PL group had a mean difference of −0.300 (SE = 0.470; 95% CI [−0.609, 0.009]; *p* = 0.057). For swimming training, the CR group showed a mean difference of −0.667 (SE = 0.285; 95% CI [−1.267, −0.066]; *p* = 0.032), while the PL group showed a mean difference of 0.400 (SE = 0.270; 95% CI [−0.170, 0.970]; *p* = 0.157). For energy intake, the CR group had a mean difference of 9.222 (SE = 29.093; 95% CI [−52.159, 70.604]; *p* = 0.755), whereas the PL group had a mean difference of −142.800 (SE = 27.600; 95% CI [−201.032, −84.568]; *p* < 0.001).

### 3.6. Intention-to-treat analysis

ITT analysis was performed including all randomized participants and results are presented in Table 5.

**Table 5.** Intention-to-treat pre- and post-supplementation outcomes by group.

Variable	Group	Pre (Mean ± SD)	Post (Mean ± SD)	*p* value (Time × Group)	Partial η^2^ (Time × Group)
TW (m)	CR	709.40 ± 236.82	910.30 ± 377.91	0.121	0.129
	PL	745.30 ± 283.83	846.80 ± 262.14		
TTE (s)	CR	116.90 ± 41.70	143.50 ± 52.84	0.093	0.149
	PL	89.50 ± 32.97	101.50 ± 37.13		
LA (mmol/L)	CR	1.15 ± 0.47	1.14 ± 0.76	0.001	0.454
	PL	1.23 ± 1.03	2.13 ± 1.09		
Mean pCO₂ (mmHg)	CR	28.96 ± 4.32	30.34 ± 4.65	0.167	0.103
	PL	27.50 ± 5.71	27.62 ± 5.84		
RPE-BH	CR	16.10 ± 2.64	16.20 ± 3.26	0.795	0.004
	PL	16.10 ± 2.02	16.10 ± 2.13		
RPE-MF	CR	13.80 ± 2.35	13.60 ± 3.57	0.210	0.086
	PL	13.10 ± 2.56	14.00 ± 2.98		
TBM (kg)	CR	87.69 ± 7.56	87.77 ± 7.42	0.861	0.002
	PL	83.67 ± 15.25	83.67 ± 14.77		
FFM (kg)	CR	61.56 ± 4.30	62.07 ± 4.43	0.092	0.150
	PL	58.10 ± 9.09	57.91 ± 8.78		
FM (kg)	CR	22.76 ± 4.99	22.35 ± 4.74	0.068	0.173
	PL	22.55 ± 8.44	22.64 ± 8.33		

A post-hoc analysis was conducted to examine differences in LA between the groups. It indicated a significant effect for the PL group, Wilks' *Λ* = 0.381, F(1, 18) = 29.224, *p* < 0.001, η^2^ = .619. The mean difference for PL was 0.900 (SE = 0.166), with a 95% confidence interval ranging from 0.550 to 1.250. In contrast, the CR group did not show a significant difference, Wilks' *Λ* = 1.000, F(1, 16) = 0.004, *p* = 0.953, η^2^ < 0.001, with a mean difference of −0.010 (SE = 0.166; 95% CI [−0.360, 0.340]).

## 4. Discussion

The present study provides novel evidence that a 7-day creatine monohydrate loading protocol enhances muscular performance during voluntary BH exercise performed on a bicycle ergometer, a protocol designed to mimic the metabolic demands and intensity of maximal dynamic BH dive with fins. The observed benefits are consistent with the established role of creatine in supporting rapid ATP re-synthesis through the phosphocreatine system and contributing to intracellular buffering capacity, which may be particularly relevant during BH exercise, when limited oxygen availability increases reliance on anaerobic energy pathways. Although both groups improved performance, as reflected by increases in TW and TTE, the larger effect sizes observed in the CR group suggest that creatine supplementation may provide an additional performance benefit beyond familiarization with the testing procedures or placebo-related effects.

The performance improvements observed in TW suggest that elevated phosphocreatine stores may contribute to meeting the energetic demands of the BH exercise task. However, it should be acknowledged that although creatine plays a key role in buffering ATP demand, it does not represent a sustainable long-term energy source. Consequently, the translation of these results to elite BH divers, who perform prolonged BH dives and cover long distances, remains uncertain.

Overall, LA values observed in the study were relatively low and a definite causal relationship cannot be established. Selective peripheral vasoconstriction may have contributed to a delayed clearance of lactate from the working musculature [6], thereby partially explaining the low measured values. Furthermore, adapted BH divers appear to exhibit physiological adaptations that enable lactate metabolism under hypoxic conditions [5,14]. The relative contribution of these mechanisms, as well as their potential interaction, remains to be fully elucidated.

Moreover, statistical analyzes revealed a large effect size in the difference in LA between CR and PL groups. Post hoc comparisons confirmed that LA increased significantly only in the PL group, while the change in the CR group was not statistically significant. This pattern suggests that, although LA increased following supplementation in both groups, the increase was attenuated in the CR group. Nevertheless, given the altered circulation and delayed lactate appearance during BH exercise, this finding should not be interpreted as direct evidence of reduced glycolysis, improved energy utilization, or delayed acidosis. An alternative explanation may involve alterations in lactate kinetics, including its production, appearance, and clearance.

In addition to higher improvements in TW and TTE observed in the CR group and the smaller lactate rise indicates that participants were able to perform more work for a longer duration while maintaining lower LA. In contrast, the PL group exhibited a larger increase in lactate with smaller improvements in performance. Thus, a mechanistic explanation would be that creatine supplementation enhanced the capacity to sustain higher workloads before reaching fatigue.

Despite inconsistencies in the literature, some strength–endurance protocols have shown improvements in TW [15–18], while others have reported an improved lactate threshold or lower post-exercise lactate levels following creatine supplementation [19–21]. Oliver et al. (2013) additionally noted that TTE approached a significant increase. TTE was significantly increased after creatine supplementation in another study employing endurance exercise with alternating intensity [22]. Other studies have shown that creatine reduced oxygen consumption during submaximal endurance exercise [23–25]. None of these studies incorporated BH into the protocol, making direct comparisons difficult. Still, it should be noted that various studies have failed to demonstrate improvements in TW, TTE, and LT in endurance-type exercise protocols [7].

The overall mean pCO₂ values observed after the BH protocol were relatively low. A likely explanation for this is delayed analyzer sensitivity, as well as the influence of dead space air. During exhalation, the initial portion of expired air originates from the anatomical dead space (e.g. trachea), where no gas exchange occurs. This air contains lower CO₂ concentrations than alveolar air, which can dilute the measured pCO₂ on the first exhale. To account for this, all breaths were considered until the CO₂ peak was reached. Therefore, the absence of clear differences in CO₂ production between groups should be interpreted in light of these technical constraints, while also considering that creatine supplementation may not substantially affect CO₂ production, as the phosphocreatine system provides only a limited energy reserve and may not contribute sufficiently to produce a measurable change in CO₂ production.

No statistically significant differences were observed in mean pCO₂ values, with both groups showing only a slight increase, likely reflecting improvements in performance. It should be noted that CO₂ production is influenced by variables such as TTE, TW, and ergometer resistance, which were not accounted for in this analysis. The results indicate that creatine supplementation did not provide a measurable benefit in this context. This may be due to the insensitivity of the measurement, the presence of confounding variables, a relatively small sample size, or the fact that creatine is not a primary or sustainable source of energy and it may only partially reduce reliance on glycolytic pathways, contributing modestly to overall energy metabolism.

From a theoretical perspective, creatine's contribution to anaerobic alactic energy production and its buffering capacity can reduce reliance on anaerobic glycolysis, thereby delaying associated decline in intramuscular pH and CO₂ production [1]. By decreasing the relative amount of hydrogen ions generated during muscle activity, phosphocreatine re-synthesis may also slightly delay the central drive to breathe, as hydrogen ions are the primary determinant of respiratory drive under normal conditions [26]. Additionally, because severe acidosis in fast-twitch fibers can contribute to force and power fatigue during exercise [27], a delayed drop in intramuscular pH may also attenuate perceived muscular fatigue. Collectively, these mechanisms provide a physiological rationale for our hypothesis that creatine supplementation would improve both RPE-BH and RPE-MF.

In the present study, RPE-BH component remained stable across supplementation in both groups. In contrast, RPE-MF demonstrated a small increase with medium size effect over time in both groups, likely reflecting the greater work performed during the post-supplementation test rather than a supplement-specific effect. Taken together, the subjective fatigue data indicate that improvements in performance were followed by a similar subjective experience.

In contrast to our results, a study by Lee et al. (2020) reported significantly lower both RPE during repeated sets of resistance exercises at 70% of 1RM in the CR group compared to the PL group. While in line with our findings, Furtado et al. (2024) found no difference between creatine and placebo in RPE while observing a significant increase in TW during the strength–endurance protocol.

In practical terms, in dynamic BHD disciplines, where performance is primarily determined by the distance covered on a single breath, TW represents a key performance outcome alongside LA for a fixed distance. From pre- to post-supplementation, the CR group improved by ≈33% in TW, whereas the PL group improved by ≈14%, reflecting an approximately 144% greater improvement in the CR compared with the PL group. Notably, these improvements in the CR group occurred alongside a modest increase in LA (≈4.3%), while the PL group exhibited a substantially larger LA (≈73.2%), indicating that CR supplementation enhanced work capacity with minimal additional metabolic disturbance.

We found no significant change in either TBM or FM over time. Nevertheless, the CR and PL groups exhibited significantly different trajectories for FFM change, with the CR group showing a significant increase in FFM, accompanied by a large effect size, compared with no significant change in the PL group. Creatine loading is known increase intracellular water, potentially manifesting as an increase in FFM [1]. Thus, the observed changes in FFM may partly reflect fluid shifts and measurement variability rather than true alterations in tissue composition and should not be interpreted as evidence of rapid changes in body composition. Furthermore, results suggested that changes in FFM may have contributed to the observed performance enhancement.

Further analyzes of training and dietary data indicated that the groups were comparable in the volume of BHD, resistance, and swimming trainings, with no significant main effect of group observed for any of these variables. Similarly, there were no significant overall changes in trainings over time. However, significant Group × Time interactions were observed for resistance and swimming training, indicating different trajectories between groups. Specifically, resistance training sessions increased in the CR group but decreased in the PL group, whereas swimming sessions decreased in the CR group but increased in the PL group. For energy intake, a significant Group × Time interaction was observed, indicating that energy intake remained relatively stable in the CR group but decreased in the PL group.

These differences in trainings and energy intake therefore may have contributed to the observed changes in body composition. Energy intake increased by 9 kcal/day in the CR group and by 143 kcal/day in the PL group, resulting in a 152 kcal/day greater reduction in the PL group relative to the CR group. Although the Group × Time interaction was statistically significant, this difference of approximately 152 kcal/day is unlikely to represent a physiologically meaningful degree of energy restriction. However, energy intake was assessed using a dietary recall from a single day rather than across the entire 7-day period preceding each testing visit; therefore, the reported intake may not accurately represent participants' energy intake during the preceding week. In addition, dietary recalls are inherently subject to self-reporting and recall bias. Similarly, training data were based on self-reported data without taking into account training intensity and may have also been affected by recall and reporting bias.

Finally, ITT analysis was performed to assess the robustness of the findings using all available data from the 20 participants who completed the study. It showed a significant between-group difference only for LA. Although other variables did not reach statistical significance, the effect sizes indicated some degree of between-group change, suggesting that the absence of statistical significance should be interpreted in the context of the sample size.

This study's strengths include (1) a novel and reliable BH exercise protocol individualized for each athlete, (2) a double-blind, randomized, placebo-controlled design, and (3) integration of performance outcomes, objective and subjective fatigue markers. Together, these provide a robust interpretation of supplementation effects.

Several limitations merit consideration. First, BH performance is influenced by both psychological tolerance to discomfort and physiological factors. Although testing was standardized to morning sessions with consistent timing, individual fluctuations in motivation may have contributed to variability. Second, the sample was predominantly male, and the menstrual cycle phase was not controlled for in female participants. Third, the group was relatively heterogeneous in terms of age, physical fitness (as assessed by both aerobic and anaerobic conditioning), and competitive level (including both national- and world-class athletes), which contributed to relatively high variability in performance metrics and the ergometer resistances used for the individual BH exercise protocol. Fourth, baseline TTE was significantly different between groups, raising the possibility that this baseline imbalance influenced the observed changes despite repeated-measures ANOVA partially accounting for this. Fifth, creatine stores were not measured directly. Sixth, despite prior familiarization with the protocol, a learning effect cannot be fully excluded. Finally, while the protocol mimicked key metabolic demands of BHD, it did not replicate the specific movement nor it captured all physiological responses elicited by water immersion. Therefore, the extent to which outcomes from a laboratory BH protocol generalize to in-water diving performance remains unknown.

From a mechanistic perspective, these findings provide support for creatine's role in enhancing intracellular buffering capacity during exercise combined with voluntary BH, whereas evidence for an effect on anaerobic alactic energy availability remains inconclusive. Proposed mechanisms of action were not directly measured and these mechanistic explanations should be interpreted as plausible hypotheses rather than demonstrated mechanisms. Further investigation on this topic should incorporate muscle biopsies to determine the extent to which creatine stores are actually utilized following maximal BH exercise.

From a practical standpoint, this study suggests that athletes participating in sports such as BHD, spearfishing, underwater rugby, synchronized swimming, and related disciplines may benefit from creatine supplementation. Additional benefits relevant to BHD may also arise from post-exercise co-ingestion of creatine and carbohydrates, which may enhance muscle glycogen re-synthesis [28]. Furthermore, benefits may be derived from its proposed effects on recovery of the acute training response, mediated by reductions in exercise-induced muscle damage, as a single BHD session has been shown to alter several serum biomarkers of skeletal muscle injury [29,30]. Moreover, creatine supplementation has been reported to increase brain creatine content, which may be of particular relevance in hypoxic conditions for maintaining cerebral energy homeostasis [31,32]. It may help preserve cognitive and neuromuscular function during demanding dives, as previous evidence has shown that 7 days of supplementation attenuated hypoxia-induced impairments in attention and increased corticomotor excitability [32].

Future studies should assess the impact of creatine supplementation on long-term training adaptations, as creatine has been suggested to increase work capacity, enhance recovery, and training adaptations [2]. Further investigation should also examine whether creatine may exert neuroprotective role relevant to BHD, particularly given the reported transient increases in a marker of brain injury following static BH [33]. Research should also evaluate the translation of these findings to real-world underwater performance, ideally using larger samples. Individual moderators—such as sex, menstrual cycle phase, training status, and baseline creatine stores—should also be considered to support the development of personalized supplementation strategies.

## Data Availability

The datasets generated and analyzed during the current study are available from the corresponding author upon reasonable request. All relevant data supporting the findings of this study are included within the article. This study was registered at ClinicalTrials.gov (Identifier: NCT07233707, https://clinicaltrials.gov/study/NCT07233707?term=load%20and%20hold). It was conducted at the Department of Nutrition and Dietetics, Harokopio University, Greece. All procedures were approved by the Research Ethics and Deontology Committee (R.E.D.C.) of Harokopio University, El. Venizelou Ave. 70, 17676 Kallithea, Attica, Greece (approval number: 3301/21-07-2025).
